# Anifrolumab in Refractory Dermatomyositis and Antisynthetase Syndrome

**DOI:** 10.1155/crrh/5560523

**Published:** 2025-04-21

**Authors:** Ryan Bonaventure Soares, Jihad Ben Gabr, Mark Ash, Gregory Hosler

**Affiliations:** ^1^Department of Medicine, SUNY Upstate Medical University, Syracuse, New York, USA; ^2^Department of Rheumatology, SUNY Upstate Medical University, Syracuse, New York, USA; ^3^Department of Dermatology, Empire Dermatology, Syracuse, New York, USA; ^4^Department of Pathology, Sonic Healthcare USA Dermatopathology Division, Austin, Texas 78727, USA

**Keywords:** anifrolumab, antisynthetase syndrome, dermatomyositis, inflammatory myopathy, interferon receptor

## Abstract

Dermatomyositis (DM) and antisynthetase syndrome are rare autoimmune disorders within the spectrum of inflammatory myopathies, typically characterized by cutaneous and muscular inflammation along with systemic manifestations. This case report highlights the evolving treatment strategies for inflammatory myopathies, with a focus on the use of anifrolumab, a type-1 interferon receptor blocker, in a 29 year-old female with refractory DM/antisynthetase syndrome. The patient presented with classic DM features, including heliotrope rash, Gottron's papules, and malar erythema, but lacked significant myopathy. Initial therapies with methotrexate and prednisone were ineffective, and her condition worsened despite adding intravenous immunoglobulin (IVIg) and tofacitinib. Following persistent disease progression, therapy was switched to a combination of IVIg, apremilast, and anifrolumab as an off-label drug for DM. Within 5 months, the patient showed significant improvement in myopathy and skin manifestations. Anifrolumab targets the interferon (IFN) axis, which plays a crucial role in DM pathogenesis. This case underscores the potential of targeted therapies like anifrolumab in managing DM, especially in cases not responsive to conventional standard of care therapies. It also highlights the need for further research into the IFN pathway as a therapeutic target in inflammatory myopathies.

**Trial Registration:** ClinicalTrials.gov identifier: NCT06455449

## 1. Introduction

Dermatomyositis (DM) and antisynthetase syndrome are rare autoimmune disorders with significant overlapping features within the broad spectrum of inflammatory myopathies. They are characterized by the typical cutaneous and muscular inflammation along with a myriad of systemic manifestations. Lack of muscle involvement can be seen in amyopathic DM. This case report underscores the evolving treatment landscape in inflammatory myopathies, particularly highlighting a therapeutic breakthrough with anifrolumab, a type-1 interferon (IFN) receptor blocker. We also discuss the rationale behind selecting anifrolumab and its mechanism of action targeting the IFN axis implicated in DM pathogenesis.

## 2. Case Presentation

**Figure 1:**
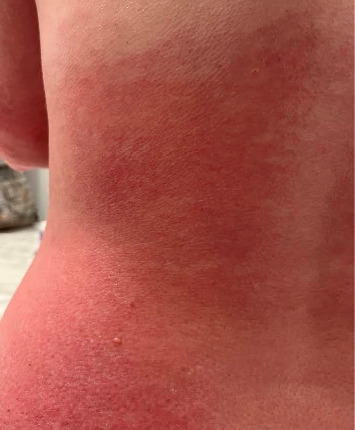
Left lower back demonstrating dermatomyositis associated rash and erythema.

We present a case of a 29-year-old female with a past medical history significant for a benign tectal glioma status postsurgery at nine years of age with VP shunt who presented with burning and pruritic erythematous papules and plaques on upper eyelids (heliotrope), scalp, extensor forearms, metacarpophalangeal (MCP) joints and proximal/distal interphalangeal joints (PIP/DIP; Gottron's papules), abdomen, lateral hips (Holster sign), malar erythema without sparing of the nasolabial fold, erythematous rash on the back (Figure 1) and periungual dilated capillaries with hemorrhage and capillary dropout along with ragged cuticles. No apparent inciting factors were identifiable. Initially this started with eyelid involvement months prior to the spread of the rash. At first presentation, she denied any systemic symptoms (specifically, patient denied any weakness, shortness of breath, or dysphagia). Examination revealed normal strength, normal lung sounds, and normal vitals (including saturating well on room air).

Laboratory studies revealed anemia, with a hemoglobin of 9.5 g/dL (reference: 11.5–15.5 g/dL), a normal white cell count and platelets. AST/ALT were mildly elevated at 54/51 U/L, respectively (reference: < 33 U/L). She had an elevated ESR of 41 mm/hr (reference: < 20), and a normal CRP of < 3 mg/dL (reference: < 8). Beta HCG was negative. Her immunology was significant for a positive ANA (1:640, speckled), normal C3 (26 mg/dL; reference: 10–40) and C4 (114 mg/dL; reference 90–180), and was negative for dsDNA, anti-Sm, rheumatoid factor, cardiolipin antibodies, anti-histone, anti-SS-A/SS-B, anti-SCL 70, anti-centromere, anti-RNP/U1-RNP, anti-cardiolipin, and anti-Jo1. Infective workup for HIV, hepatitis C, hepatitis B was negative. Aldolase was within normal limits (9.5 U/L; reference: 3.3–10.3 U/L). Serum protein and immunofixation electrophoresis did not show paraproteins. The results of a comprehensive MyoMarker panel are illustrated in Table 1.

**Figure 2:**
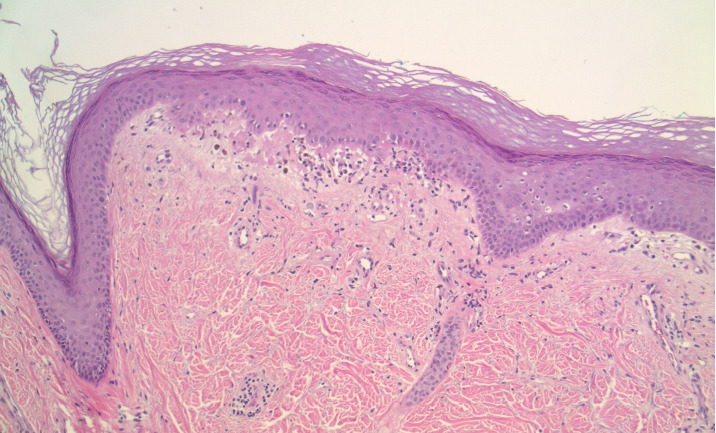
Skin biopsy showing interface dermatitis with basement membrane thickening and sparse lymphocytic infiltrate.

Double positivity of PL-7 and PL-12 is a rare entity that was present in the evaluation of this patient. As for the methodology, ELISA with radioimmunoprecipitation assay (RIPA) gel radiography was used. Muscle biopsy was not performed due to presence of diagnostic criteria for DM and lack of clinical myopathy at first presentation. Skin biopsy (Figure 2) showed interface dermatitis (basilar vacuolopathy and dyskeratosis) with basement membrane thickening and sparse lymphocytic infiltrate around superficial vessels, which may be seen with both DM and lupus erythematosus (histology alone cannot be used to distinguish between the two).

Based on her clinical picture, positive immunology for PL-7/PL-12 antibody (negative anti-MDA5 and Mi-2), and a skin biopsy showing interface dermatitis, a diagnosis of antisynthetase syndrome was made. Pulmonary evaluation with pulmonary function test with diffusion capacity did not show any evidence of interstitial lung disease (ILD). Based on her diagnosis of DM and a strong family history of malignancy (metastatic cutaneous squamous cell carcinoma in her father and lymphoma, renal cell cancer and breast/uterine cancers in her grandfather, paternal uncle and grandmother, respectively), a screening PET scan was performed, which was negative for malignancy.

Beyond topical steroids, she was initially started on methotrexate 5 mg once weekly (later increased to 15 mg weekly), folic acid 1 mg daily and prednisone 60 mg daily after diagnosis. However, prior to lung imaging, methotrexate was discontinued after a few weeks to ensure no methotrexate-associated acute pneumonitis was present at the time of imaging. Additionally, the patient preferred to avoid intrauterine device placement or oral birth control and was therefore started instead on azathioprine (max dose 75 mg twice a day) with later addition of IVIg every 4 weeks as she remained refractory and developed subtle myopathy.

Due to lack of response, progressive myopathy, and inability to escalate IVIg beyond every 3 weeks due to insurance coverage issues, she was switched to tofacitinib and IVIg. Her treatment course with tofacitinib was complicated with recurrent shunt infections requiring hospital admission for intravenous (IV) antibiotics, chronic antibiotic suppression and VP shunt revision surgeries. Furthermore, she had minimal improvement as she developed dysphagia and a 50-pound weight-loss, warranting placement of a GJ tube. Her proximal myopathy continued to progress, affecting her activities of daily life, and subsequently, quality of life. A thorough gastroenterology evaluation of her esophagus via an EGD was done, which showed no abnormalities related to motility and sphincter function. She did, however, have diffuse esophageal candidiasis which was treated with antifungals. Repeat screening CT chest, abdomen and pelvis were also negative for malignancy.

After resolution of her infection and removal of her VP shunt, therapy was switched to IVIg, apremilast, and anifrolumab, a type-1 IFN receptor (IFNAR1) blocker.

**Figure 3:**
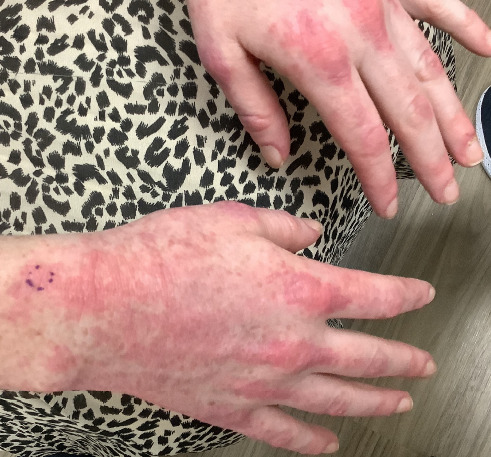
Preanifrolumab—dorsal aspect on the hands showing the classical dermatomyositis rash.

**Figure 4:**
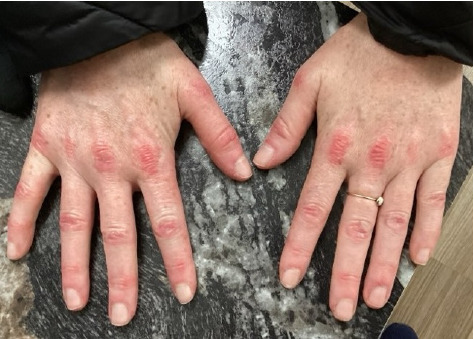
Postanifrolumab—appropriate response and improvement in the dermatological signs after anifrolumab therapy.

**Figure 5:**
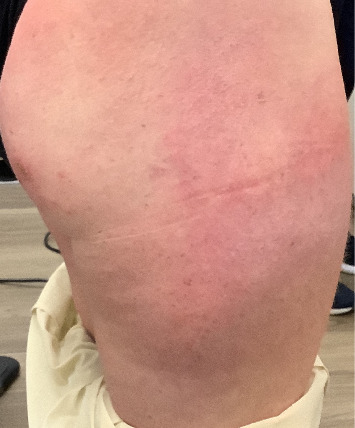
Preanifrolumab—lateral right hip demonstrating dermatomyositis associated skin rash and erythema.

**Figure 6:**
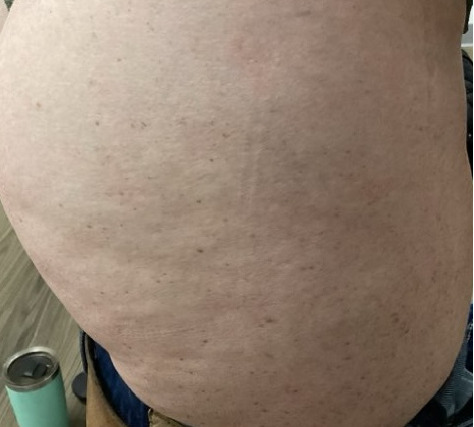
Postanifrolumab—lateral right hip demonstrating clinical response to anifrolumab with resolution of the rash.

Within 5 months, she reported immense subjective improvement in her myopathy and skin rash, and she was completely weaned off steroids. She was also able to tolerate a regular diet with near-complete resolution of her dysphagia, allowing for GJ tube removal and restoration of her baseline weight. Clinical improvement has been illustrated in the images below, which portray dermatological findings pre- and postanifrolumab treatment (Figures 3, 4, 5, 6).

## 3. Discussion

Idiopathic inflammatory myopathies consist of a group of heterogenous disorders which includes DM, polymyositis, immune-mediated necrotizing myopathy (IIM), inclusion body myositis (IBM) and overlap myositis syndromes [1]. Idiopathic inflammatory myopathies stem from imbalances in cytokine signaling [2] and T-cell dysregulation, creating a proinflammatory environment which fuels the disease process, including IFNs, TNF-alpha, janus kinases, IL-6, etc.

Antisynthetase syndrome is a rare disorder, characterized by autoantibody production against aminoacyl-transfer ribonucleic acid synthetases, including but not limited to Jo1, PL7, and PL-12. Among the autoantibodies, anti-Jo1 is the most prevalent. Clinically, antisynthetase syndrome is more likely to have associated ILD and nonerosive arthritis and often has typical findings seen in DM and polymyositis, such as myopathy, Raynaud's phenomenon and DM skin changes such as heliotrope rash, violaceous rash, V-signs and shawl sign [3, 4]. Increased prevalence of ILD has been reported in antisynthetase syndrome with PL-7/PL-12 positivity as compared to anti-Jo-1.

Classic treatment strategies for DM and antisynthetase syndrome include prednisone, methotrexate, mycophenolate, azathioprine, IVIg and lastly rituximab and cyclophosphamide. IVIg is FDA approved for use in DM, with one 16-week Phase 3 trial demonstrating its superiority as compared to placebo according to the Total Involvement Score (TIS) of at least 20, which was 79% in the treatment group versus 44% in the placebo group (95% CI 17-53, *p* < 0.001) [5].

With the development of targeted antibody and small molecule therapies, treatment has evolved, especially for patients with multisystem disease refractory to standard of care treatments. However, anti-TNF alpha agents, which were among the first developed (multiple trials examined etanercept and infliximab) failed to meet their primary endpoints, with an increased incidence of adverse events being reported in treatment groups [6]. A major setback of the infliximab trials was the development of DM flares in patients who were being treated for other conditions, which was due to the activation of the IFN axis [7]. Conversely, published case reports have also highlighted promising efficacy of tofacitinib in anti-MDA5 positive DM patients with ILD [8]. Apremilast has also demonstrated so me efficacy in DM through a possible interference in the T-helper (Th1 and Th2) response [9].

In our case, the patient did not have evidence of ILD but developed myopathy and dysphagia with refractory disease flares despite standard of care therapies, including methotrexate, azathioprine, IVIG, and a tofacitinib. Consequently, given the similarity in cytokine activation between lupus and DM, anifrolumab was added to IVIg and apremilast therapy in this patient.

There are two main IFN signaling classes, type 1 and type 2, both of which activate common and distinct STAT complexes, which then subsequently control or regulate target gene transcription. As the pathophysiology of DM involves an increase in type-1 IFN signaling, as a last resort, we decided to use anifrolumab, a IFNAR1-blocking antibody, as an off-label targeted approach which proved extremely helpful in controlling her disease activity. Studies have shown that increased type 1 IFN signature in DM patients, correlated with disease activity [10]. Antisynthetase syndrome typically has a lower IFN signature as compared to DM or SLE, but can vary individually. In a Phase 1b randomized control trial, a similar drug, sifalimumab which is another IFNAR1-blocking agent demonstrated greater neutralization of IFN signature, in patients with > 15% Manual Muscle Testing (MMT) improvement in both muscle and blood [11]. IFN 1 signature in the blood can potentially play a useful role as a marker for treatment response, as it has been shown to be downregulated after effective therapy [12]. Patients with DM who are MDA5 antibody negative are noted to have a higher IFN 1 signature in muscle [13], which may explain the rapid improvement in myopathic symptoms in our patient after anifrolumab therapy. Anifrolumab has been FDA approved for use in SLE, while sifalimumab is still in Phase 2 trials for SLE [14, 15]. However, these drugs are currently not FDA approved for DM or antisynthetase syndrome. To our knowledge, there is only one phase III clinical trial (JASMINE), started in June 2024, designed to investigate the efficacy and safety of subcutaneous anifrolumab (vs. placebo) as an add-on to the standard of care therapy in patients with DM/PM [16].

## 4. Conclusions

In conclusion, the presented case underscores the complexity and challenges in managing DM, particularly in the context of antisynthetase syndrome and added resistance to current standard of care therapies. We highlight the clinical efficacy of anifrolumab in this patient, which proved to be extremely useful in controlling the disease. This case highlights the evolving landscape of treatment options for inflammatory myopathies, emphasizing the potential role of targeted biologics like anifrolumab in managing complex cases unresponsive to conventional therapies. We hope this report would encourage further studies and clinical trials, especially exploring the IFN axis which has shown to be a major potential therapeutic target in these patients.

## Figures and Tables

**Table 1:** Comprehensive MyoMarker immunology panel.

Test	Value	Reference range
PL-7	Positive	Negative
PL-12	Positive	Negative
EJ	Negative	Negative
OJ	Negative	Negative
SRP	Negative	Negative
Mi-2	Negative	Negative
TIF1 gamma	< 20	< 20
MDA5	< 20	< 20
NXP-2	< 20	< 20
Anti-PM/Scl-100	< 20	< 20
Fibrillarin (U3 RNP)	Negative	Negative
U2 snRNP	< 20	< 20
Ku	Negative	Negative

## Data Availability

The data that support the findings of this study are available from the corresponding author upon reasonable request.

## References

[1] Lundberg I. E., Fujimoto M., Vencovsky J. (2021). Idiopathic Inflammatory Myopathies. *Nature Reviews Disease Primers*.

[2] Carolina Londe A., Fernandez-Ruiz R., Rogério Julio P. (2023). Type I Interferons in Autoimmunity: Implications in Clinical Phenotypes and Treatment Response. *Journal of Rheumatology*.

[3] Cojocaru M., Cojocaru I. M., Chicos B. (2016). New Insights into Antisynthetase Syndrome. *Maedica (Bucur)*.

[4] Hum R. M., Lilleker J. B., Lamb J. A. (2024). Comparison of Clinical Features Between Patients With Anti-Synthetase Syndrome and Dermatomyositis: Results From the MYONET Registry. *Rheumatology*.

[5] Aggarwal R., Charles-Schoeman C., Schessl J. (2022). Trial of Intravenous Immune Globulin in Dermatomyositis. *New England Journal of Medicine*.

[6] van den Hoogen L. L., van Laar J. M. (2020). Targeted Therapies in Systemic Sclerosis, Myositis, Antiphospholipid Syndrome, and Sjögren’s Syndrome. *Best Practice & Research Clinical Rheumatology*.

[7] Dastmalchi M., Grundtman C., Alexanderson H. (2008). A High Incidence of Disease Flares in an Open Pilot Study of Infliximab in Patients With Refractory Inflammatory Myopathies. *Annals of the Rheumatic Diseases*.

[8] Chen Z., Wang X., Ye S. (2019). Tofacitinib in Amyopathic Dermatomyositis–Associated Interstitial Lung Disease. *New England Journal of Medicine*.

[9] Bitar C., Maghfour J., Ho-Pham H., Stumpf B., Boh E. (2019). Apremilast as a Potential Treatment for Moderate to Severe Dermatomyositis: A Retrospective Study of 3 Patients. *JAAD Case Reports*.

[10] Sanner H., Schwartz T., Flatø B., Vistnes M., Christensen G., Sjaastad I. (2014). Increased Levels of Eotaxin and MCP-1 in Juvenile Dermatomyositis Median 16.8 Years After Disease Onset; Associations With Disease Activity, Duration and Organ Damage. *PLoS One*.

[11] Higgs B. W., Zhu W., Morehouse C. (2014). A Phase 1b Clinical Trial Evaluating Sifalimumab, an Anti-IFN-α Monoclonal Antibody, Shows Target Neutralisation of a Type I IFN Signature in Blood of Dermatomyositis and Polymyositis Patients. *Annals of the Rheumatic Diseases*.

[12] Walsh R. J., Sek W. K., Yao Y. (2007). Type I Interferon-Inducible Gene Expression in Blood is Present and Reflects Disease Activity in Dermatomyositis and Polymyositis. *Arthritis & Rheumatology*.

[13] Allenbach Y., Leroux G., Suárez-Calvet X. (2016). Dermatomyositis With or Without Anti-Melanoma Differentiation-Associated Gene 5 Antibodies: Common Interferon Signature But Distinct NOS2 Expression. *American Journal of Pathology*.

[14] Balogh L., Oláh K., Sánta S., Majerhoffer N., Németh T. (2024). Novel and Potential Future Therapeutic Options in Systemic Autoimmune Diseases. *Frontiers in Immunology*.

[15] Khamashta M., Merrill J. T., Werth V. P. (2016). Sifalimumab, an Anti-Interferon-α Monoclonal Antibody, in Moderate to Severe Systemic Lupus Erythematosus: A Randomised, Double-Blind, Placebo-Controlled Study. *Annals of the Rheumatic Diseases*.

[16] A Study to Investigate the Efficacy and Safety of Anifrolumab Administered as Subcutaneous Injection and Added to Standard of Care Compared with Placebo Added to Standard of Care in Adult Participants with Idiopathic Inflammatory Myopathies (Polymyositis and Dermatomyositis). *ClinicalTrials.Gov*.

